# Supplementary material for: Pipecolisporin, a Novel Cyclic Peptide with Antimalarial and Antitrypanosome Activities from a Wheat Endophytic Nigrospora oryzae
Source: Pharmaceuticals (Basel). 2021 Mar 16;14(3):268. doi: 10.3390/ph14030268 (PMC8000807; doi:10.3390/ph14030268)
Supplement: Supplementary file 1 [file pharmaceuticals-14-00268-s001.pdf]

Supporting Information

Pipecolisporin, a Novel Cyclic Peptide with Antimalarial and Antitrypanosome Activities from a Wheat endophytic *Nigrospora oryzae*

Ignacio Fernández-Pastor[1], Victor González-Menéndez[1], Frederick Annang[1], Clara Toro[1], Thomas A Mackenzie[1], Cristina Bosch-Navarrete[2], Olga Genilloud[1], Fernando Reyes[1*]

[1] Fundación MEDINA, Parque Tecnológico de Ciencias de la Salud, Avda. del Conocimiento 34, 18016, Armilla Granada, Spain.
[2] Instituto de Parasitología y Biomedicina "López-Neyra", Consejo Superior de Investigaciones Científicas (CSIC) Avda. del Conocimiento 17, 18016, Armilla, Granada, Spain.
Corresponding Author

* E-mail: fernando.reyes@medinaandalucia.es Tel: +34 958 993965.

Supporting Information Contents

Figure S1. CF-298113 Solid State Fermentation on BRFT medium after 21 days of incubation at 22 °C.

Figure S2. LC-UV (210 nm) chromatogram of pipecolisporin (**1**).

Figure S3. ESI-TOF spectrum of pipecolisporin (**1**).

Figure S4. UV spectrum of pipecolisporin (**1**).

Figure S5. ^{1}H-NMR (500 MHz, DMSO-d_6) spectrum of pipecolisporin (**1**).

Figure S6. ^{13}C-NMR (125 MHz, DMSO-d_6) spectrum of pipecolisporin (**1**).

Figure S7. HSQC (DMSO-d_6) spectrum of pipecolisporin (**1**).

Figure S8. HMBC (DMSO-d_6) spectrum of pipecolisporin (**1**).

Figure S9. COSY (DMSO-d_6) spectrum of pipecolisporin (**1**).

Figure S10. TOCSY (DMSO-d_6) spectrum of pipecolisporin (**1**).

Figure S11. NOESY (DMSO-d_6) spectrum of pipecolisporin (**1**).

Figure S12. Chromatographic profile of the standard amino acids present in pipecolisporin (**1**) derivatized with L-FDVA.

Figure S13. Chromatographic profile of the amino acids from the hydrolizate of pipecolisporin (**1**) derivatized with L-FDVA.

Figure S14. Overlay of HSQC NMR spectra of a hydrolysate of pipecolisporin (**1**) (red and blue NMR signals) and L-Ile standard amino acid (green and yellow NMR signals).

Figure S15. Overlay of HSQC NMR spectra of a hydrolysate of pipecolisporin (**1**) (red and blue NMR signals) and L-*allo*-Ile standard amino acid (green and yellow NMR signals).

Figure S16. Growth inhibition curve of pipecolisporin (**1**) against *Tripanosoma cruzi*.

Figure S17. Growth inhibition curve of benznidazole against *T. cruzi.*

Figure S18. Growth inhibition of pipecolisporin (**1**) against *Plasmodium falciparum* 3D7.

Figure S19. Growth inhibition curve of chloroquine against *P. falciparum* 3D7.

Figure S20. Growth inhibition curves of pipecolisporin (**1**) against human cell lines.

Figure S21. *Nigrospora oryzae* CF-298113. Upper surface overview of a 21 days culture after inoculation on a) Yeast-Malt medium; b) cornmeal medium.

Figure S22. Conidiogenous cells giving rise to conidia. Scale bars: a-c = 10 μm.

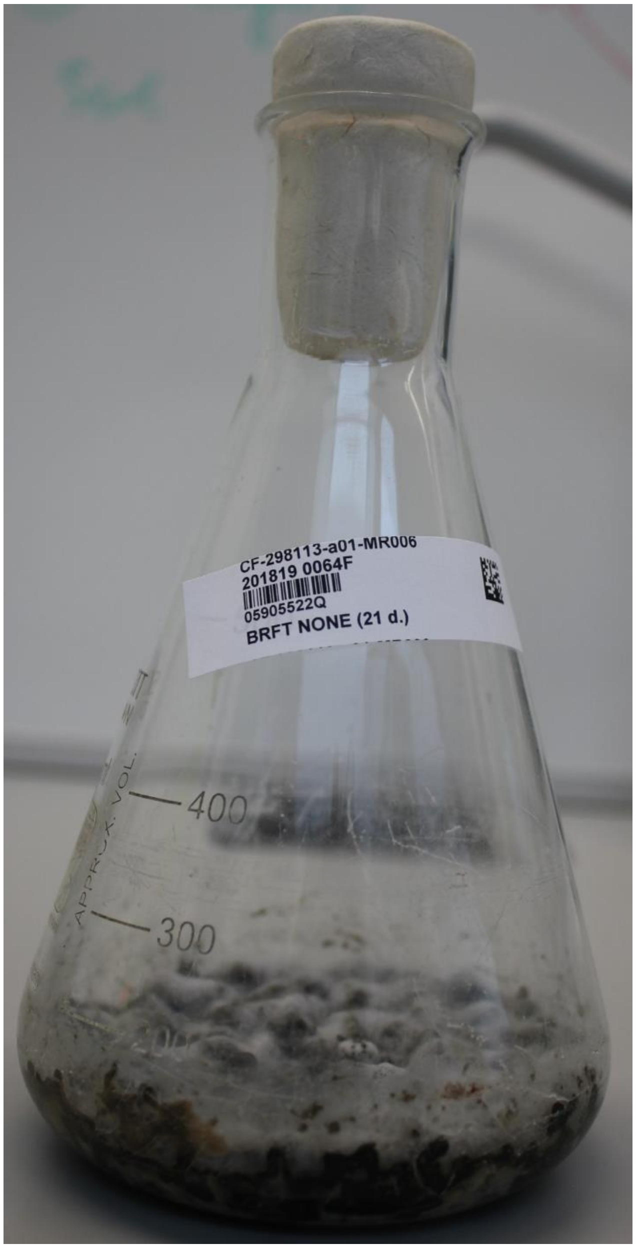

Figure S1. CF-298113 Solid State Fermentation on BRFT medium after 21 days of incubation at 22 °C.

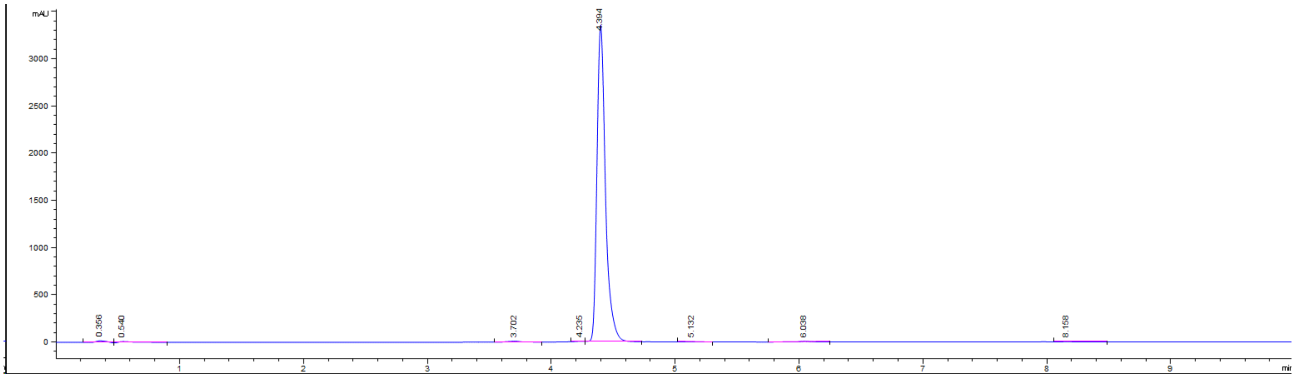

Figure S2. LC-UV (210 nm) chromatogram of pipecolisporin (**1**).

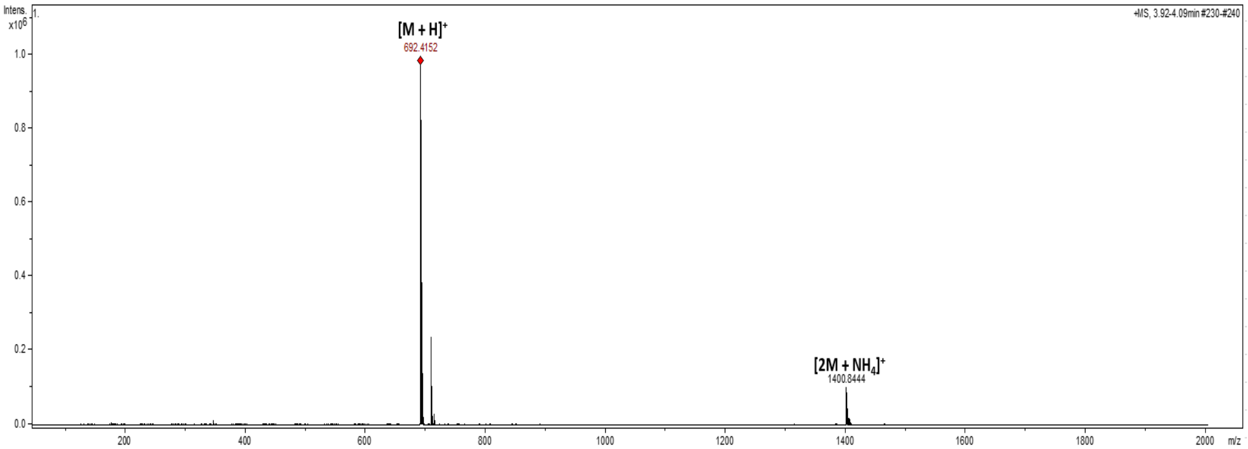

Figure S3. ESI-TOF spectrum of pipecolisporin (**1**).

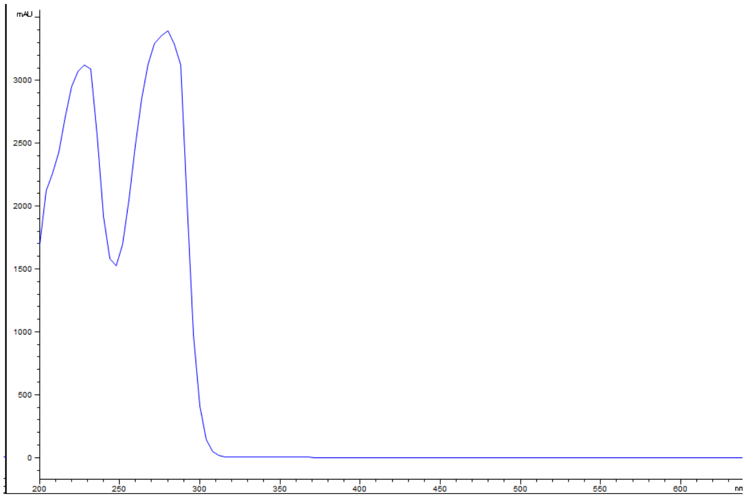

Figure S4. UV spectrum of pipecolisporin (**1**).

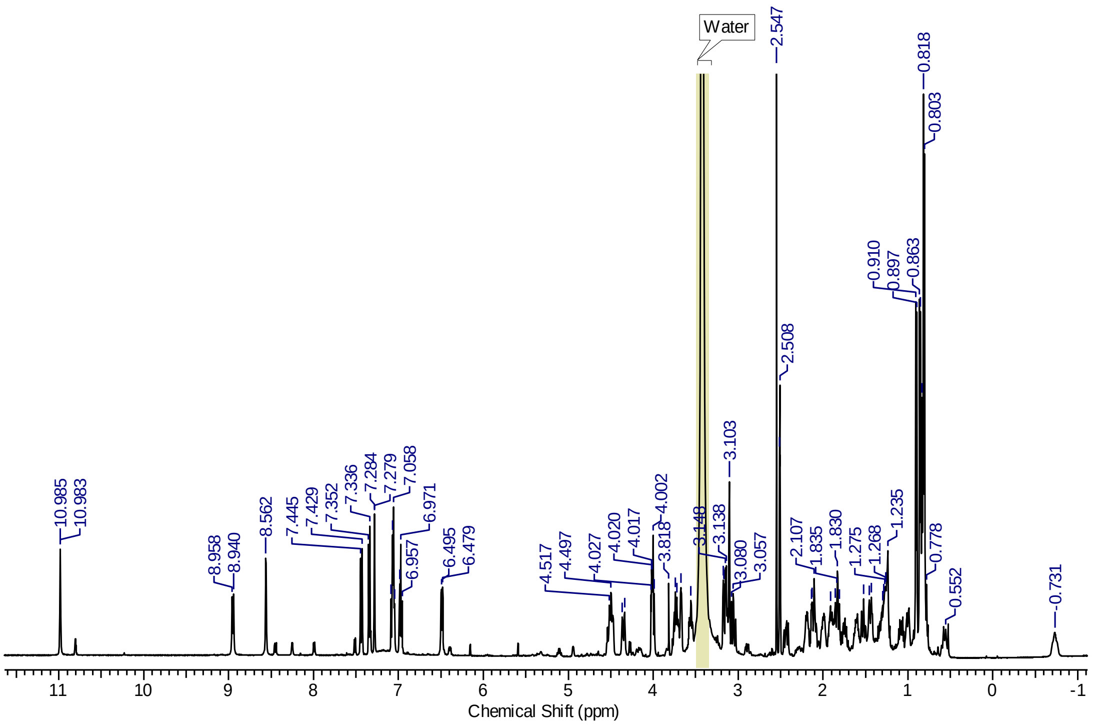

Figure S5. ^{1}H-NMR (500 MHz, DMSO-d_6) spectrum of pipecolisporin (**1**).

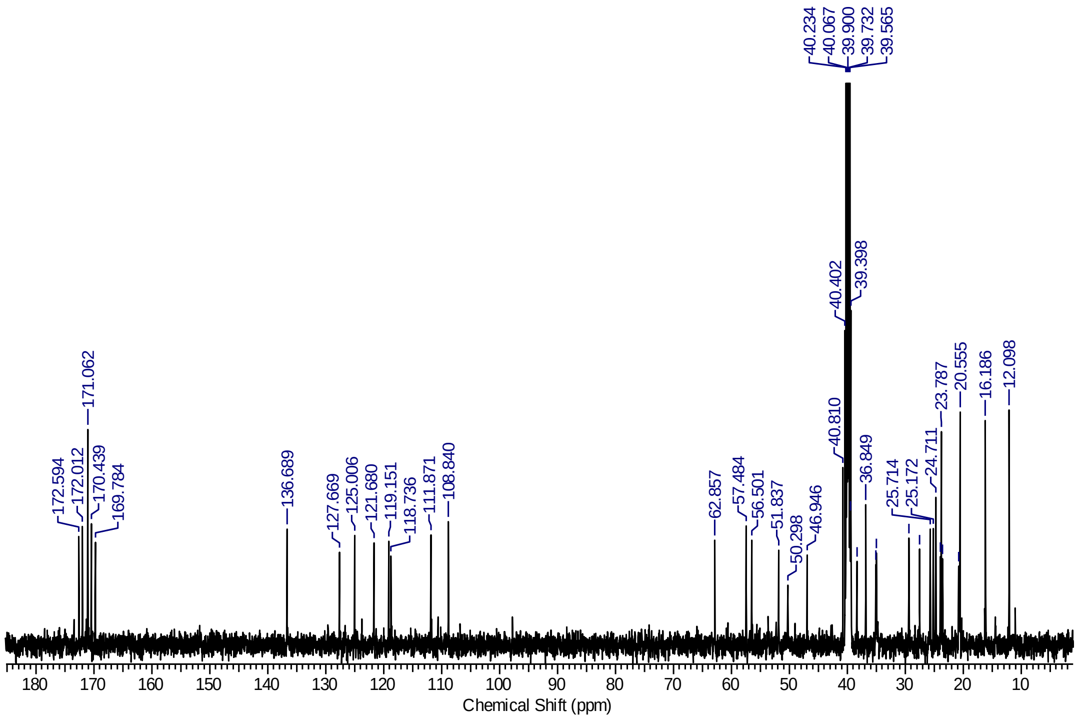

Figure S6. ^{13}C-NMR (125 MHz, DMSO-d_6) spectrum of pipecolisporin (**1**).

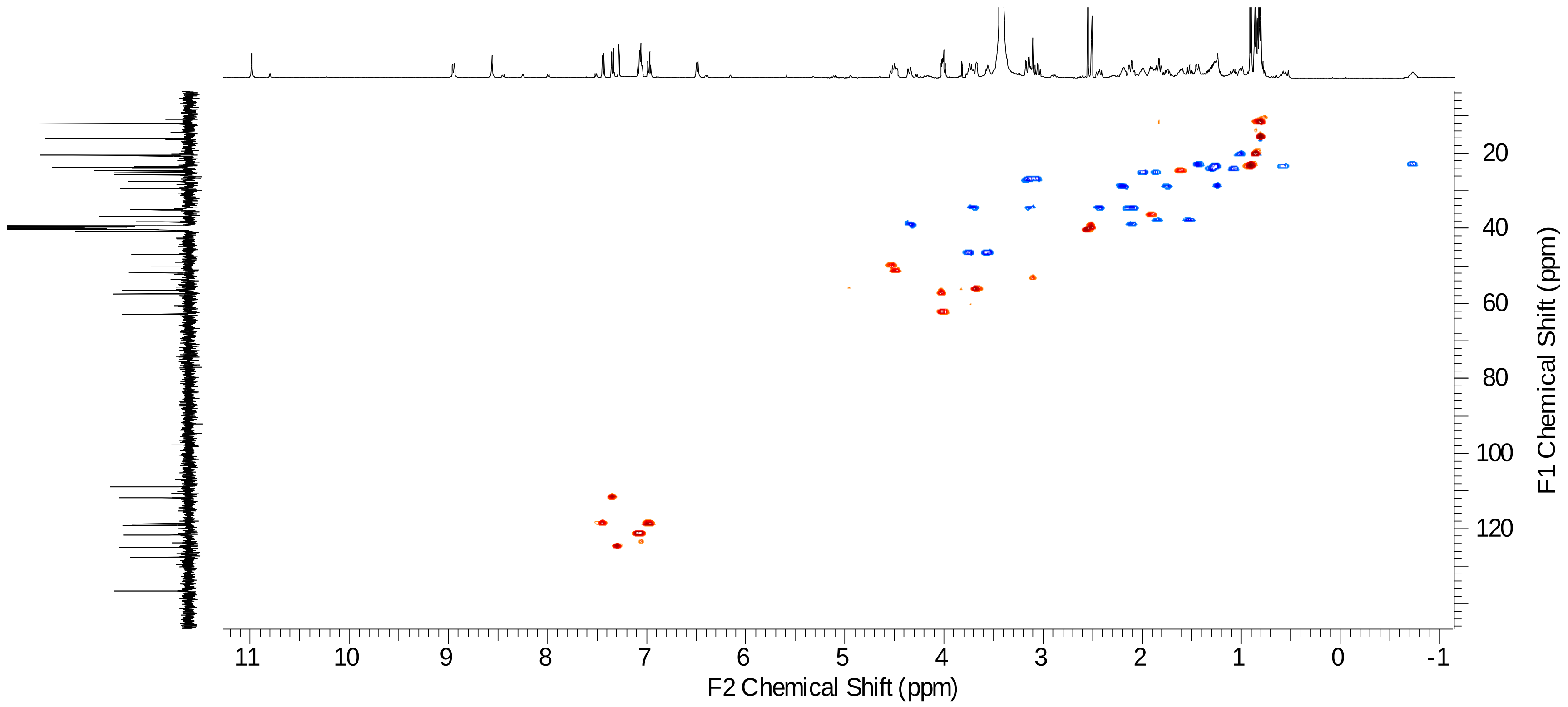

Figure S7. HSQC (DMSO-d_6) spectrum of pipecolisporin (**1**).

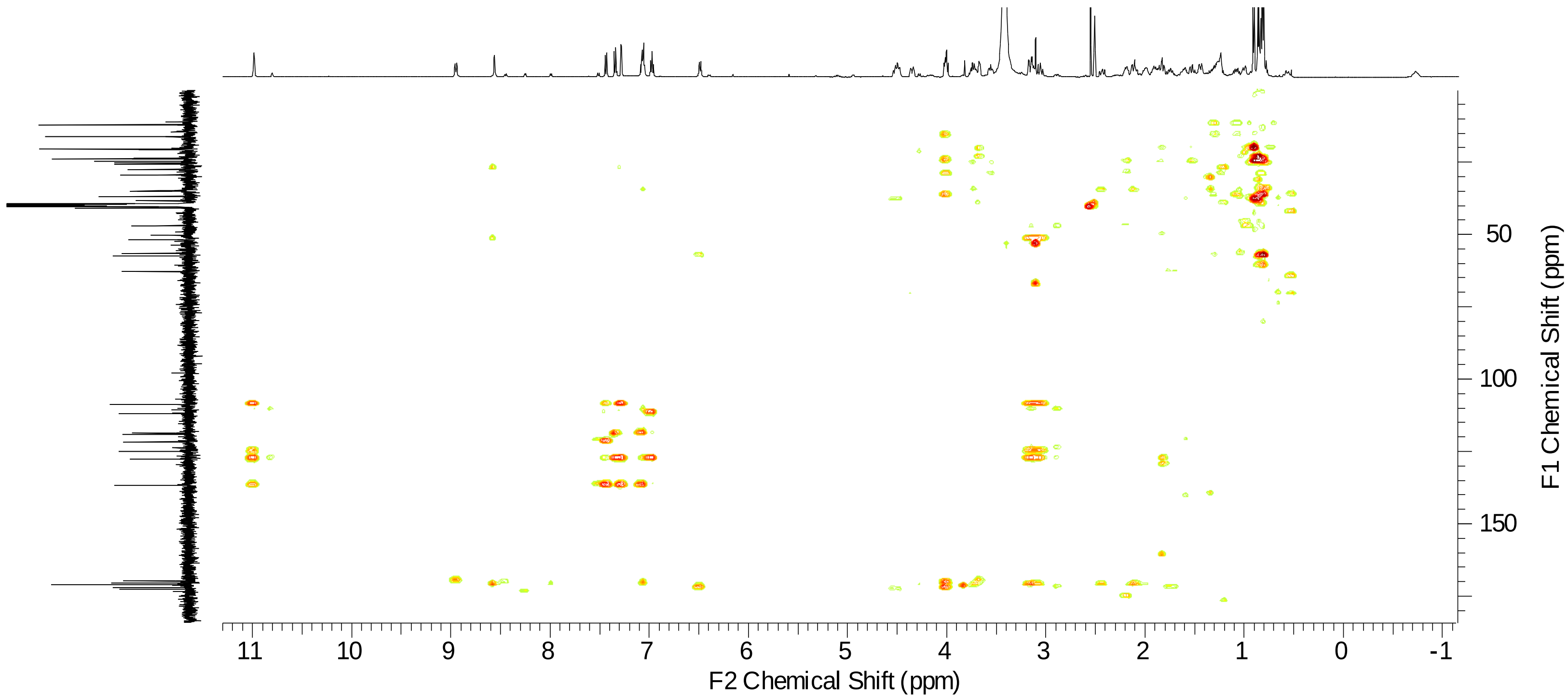

Figure S8. HMBC (DMSO-d_6) spectrum of pipecolisporin (**1**).

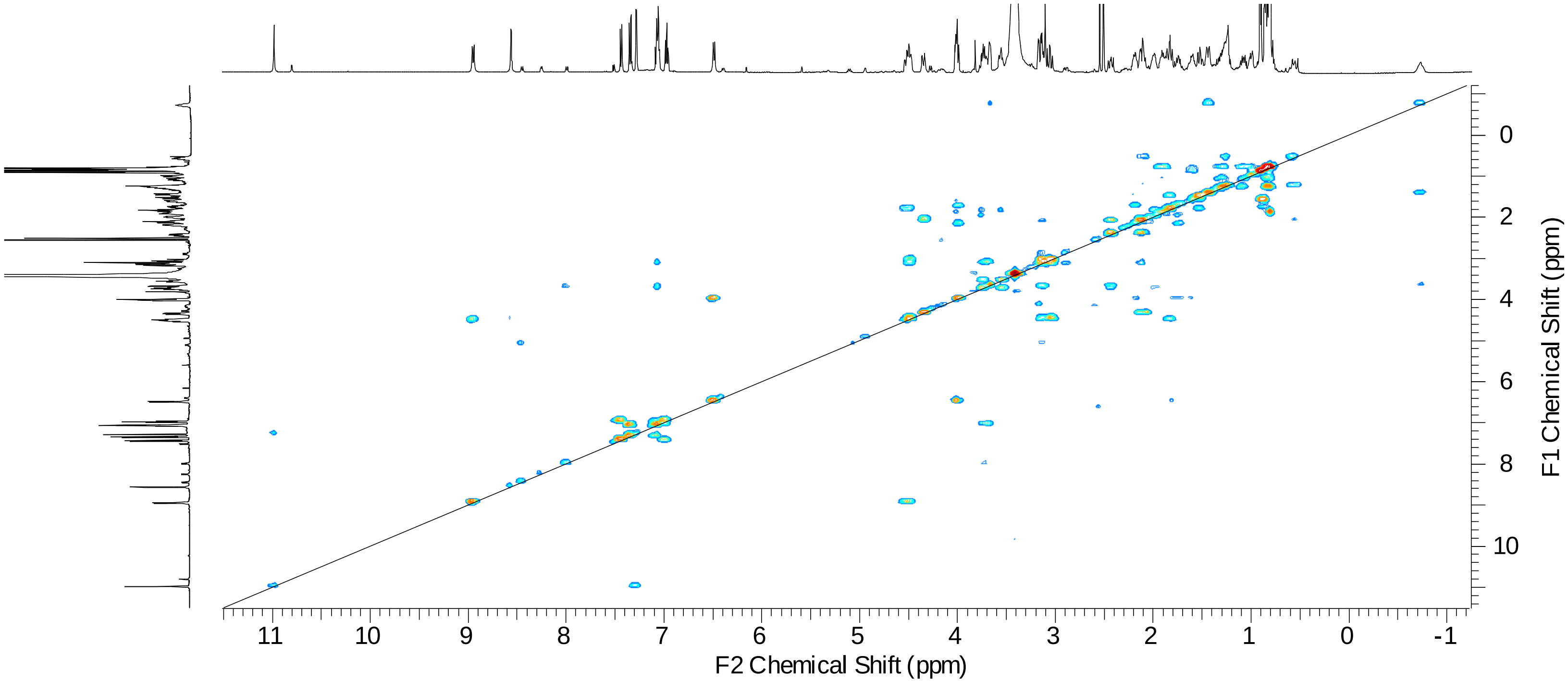

Figure S9. COSY (DMSO-*d*6) spectrum of pipecolisporin (**1**).

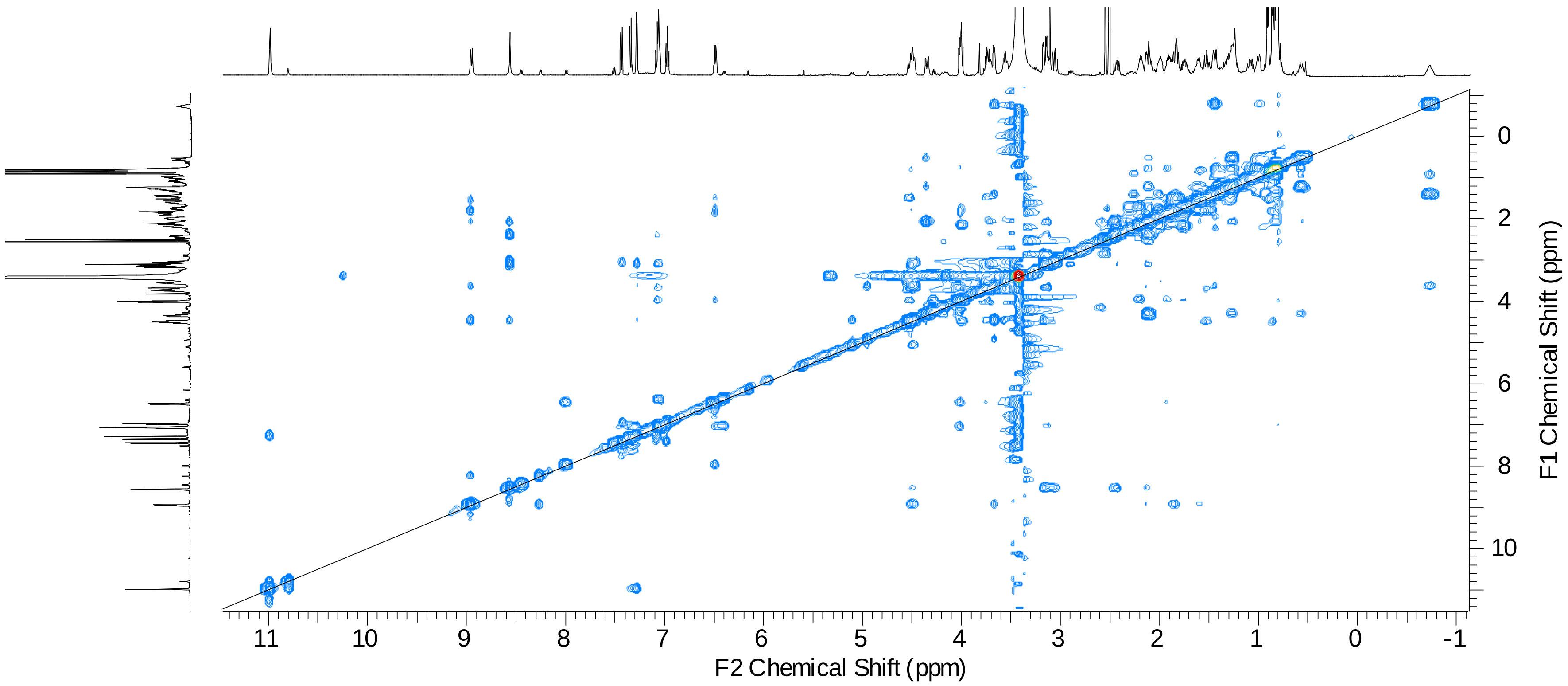

Figure S10. TOCSY (DMSO-d_6) spectrum of pipecolisporin (**1**).

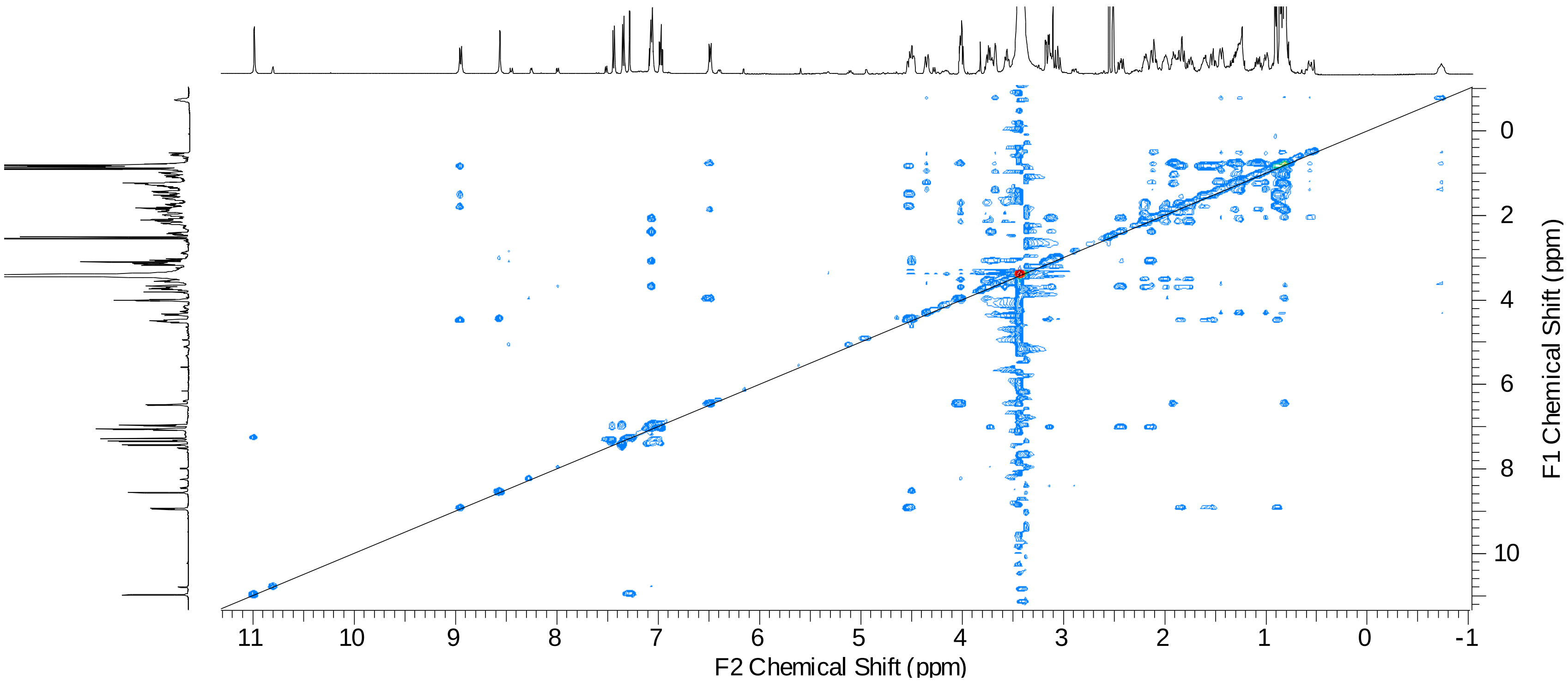

Figure S11. NOESY (DMSO-d_6) spectrum of pipecolisporin (**1**).

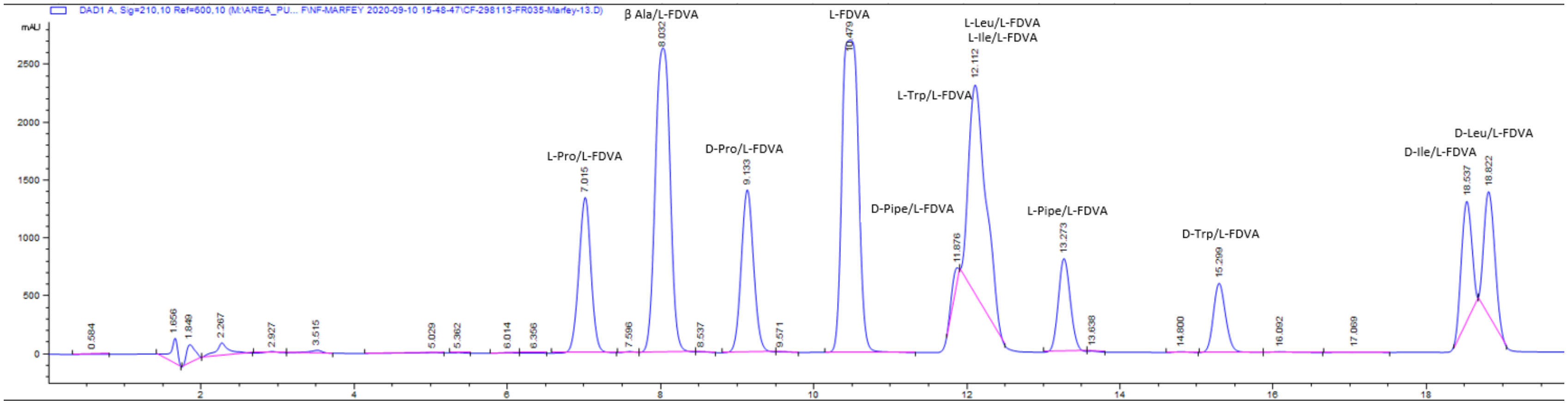

Figure S12. Chromatographic profile of the standard amino acids present in pipecolisporin (**1**) derivatized with L-FDVA.

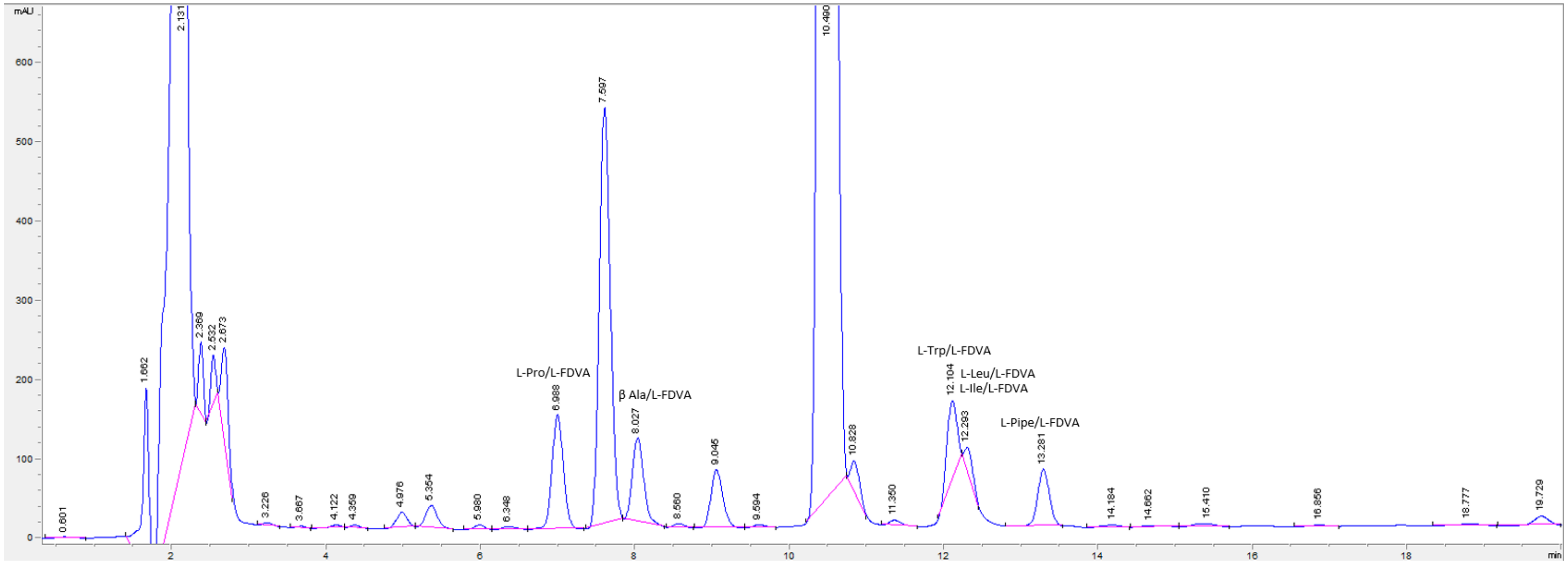

Figure S13. Chromatographic profile of the amino acids from the hydrolizate of pipecolisporin (**1**) derivatized with L-FDVA.

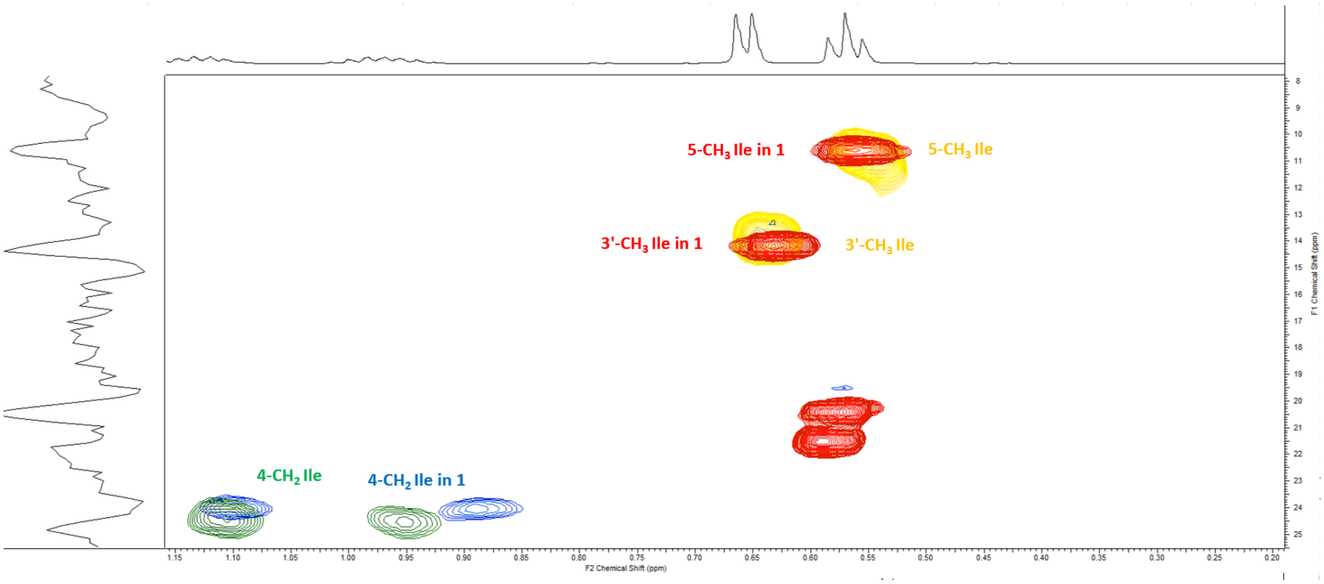

Figure S14. Overlay of HSQC NMR spectra of a hydrolysate of pipecolisporin (**1**) (red and blue NMR signals) and L-Ile standard amino acid (green and yellow NMR signals).

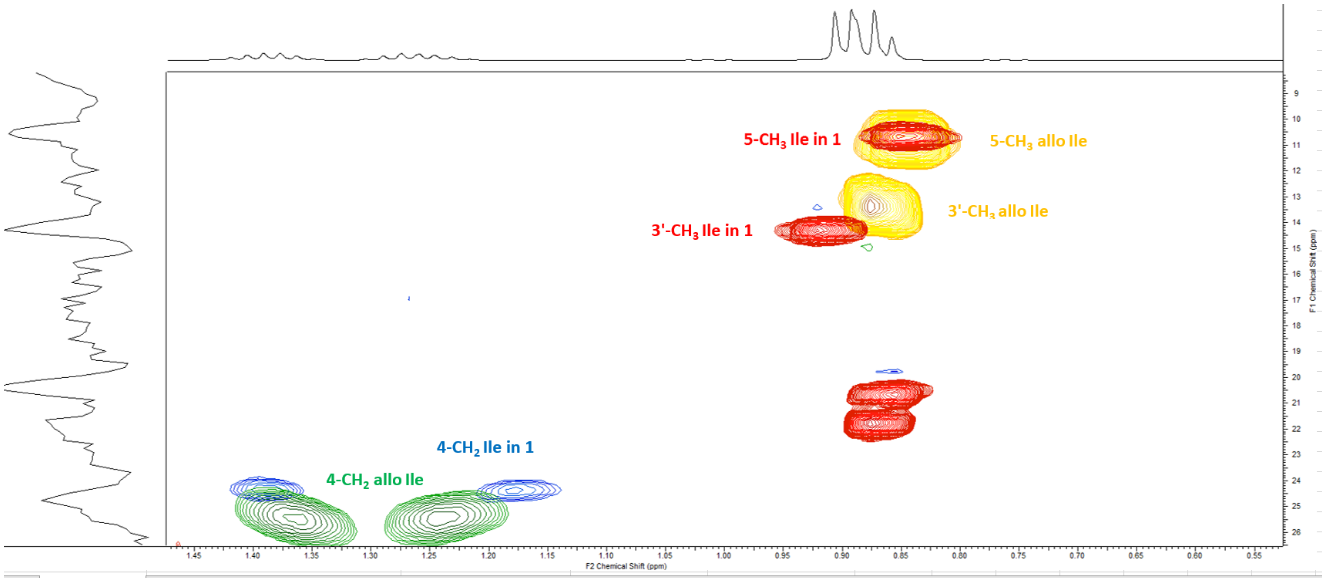

Figure S15. Overlay of HSQC NMR spectra of a hydrolysate of pipecolisporin (**1**) (red and blue NMR signals) and L-*allo*-Ile standard amino acid (green and yellow NMR signals).

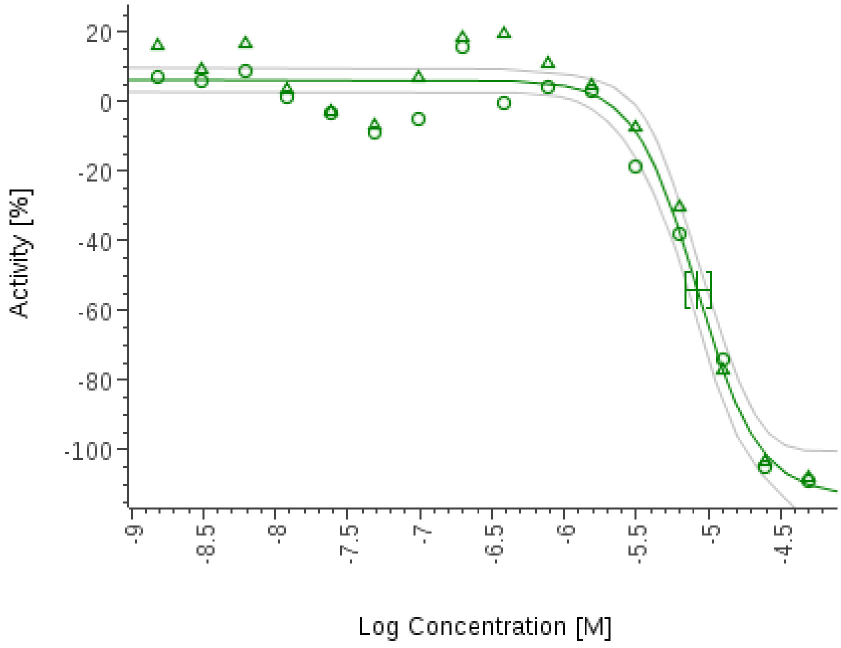

Figure S16. Growth inhibition curve of pipecolisporin (**1**) against *Tripanosoma cruzi*.

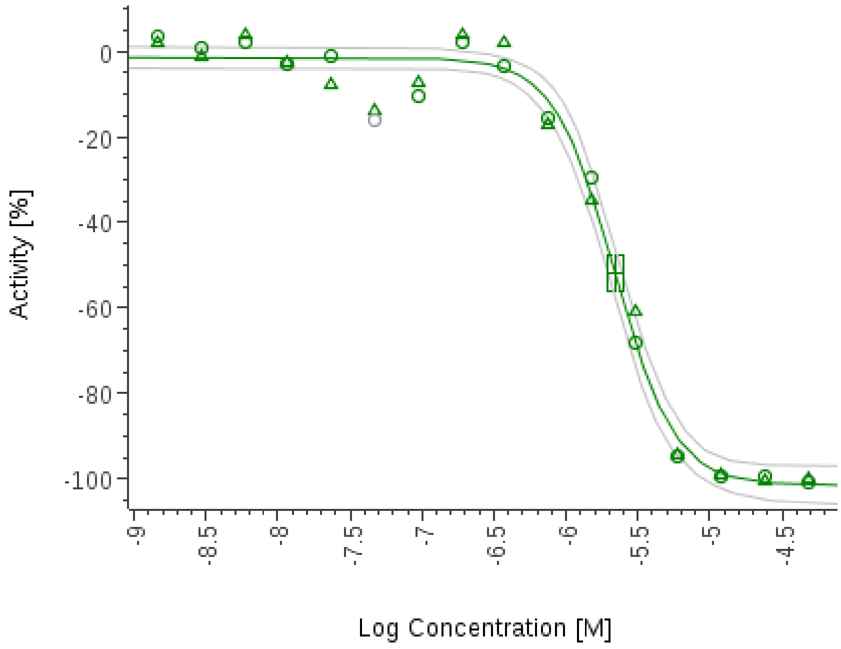

Figure S17. Growth inhibition curve of benznidazole against *T. cruzi.*

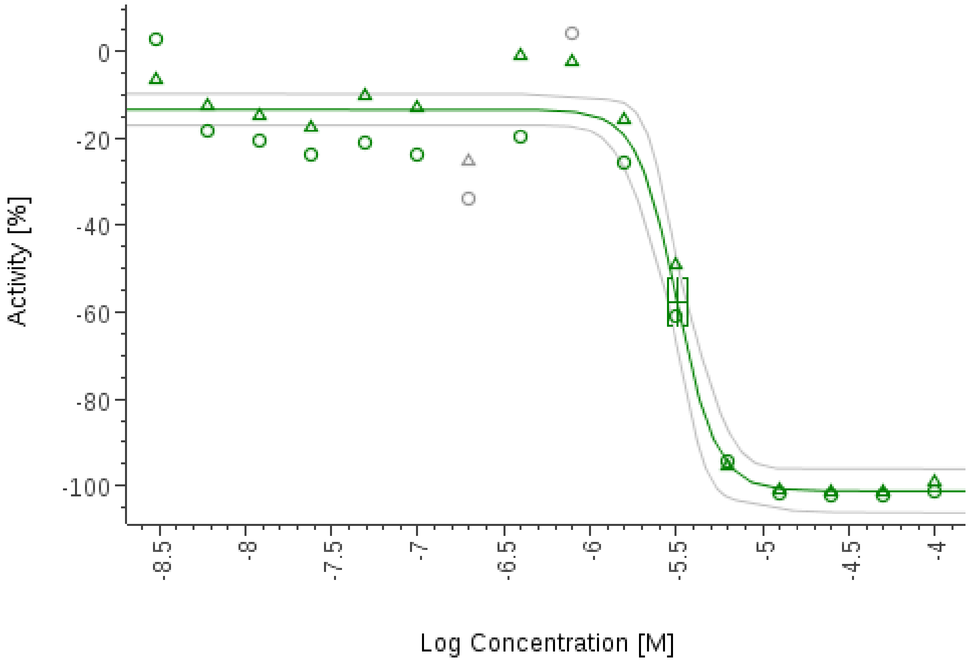

Figure S18. Growth inhibition curve of pipecolisporin (**1**) against *P. falciparum* 3D7.

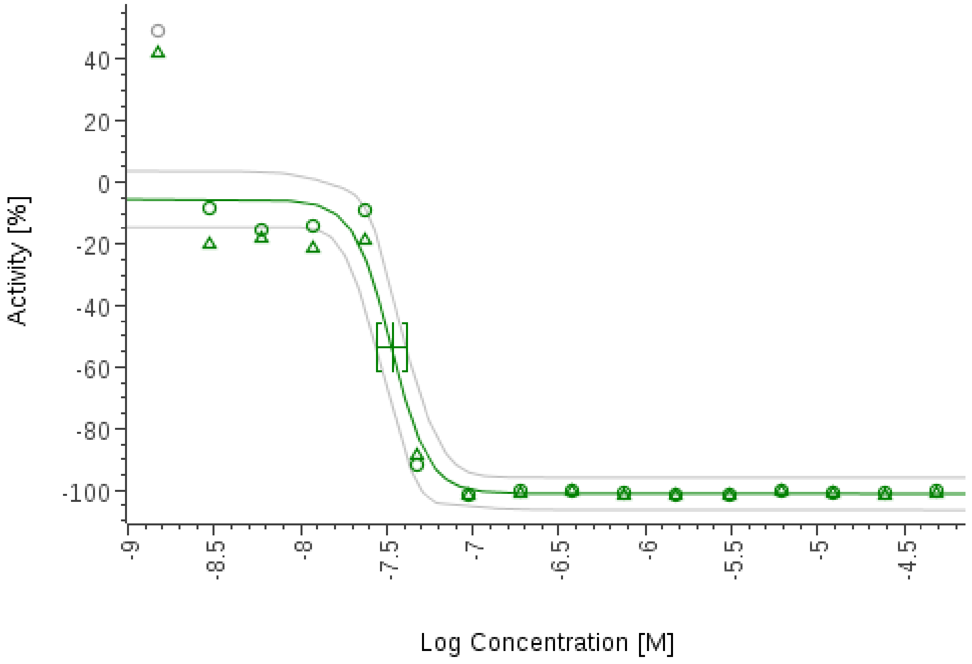

Figure S19. Growth inhibition curve of chloroquine against *P. falciparum* 3D7.

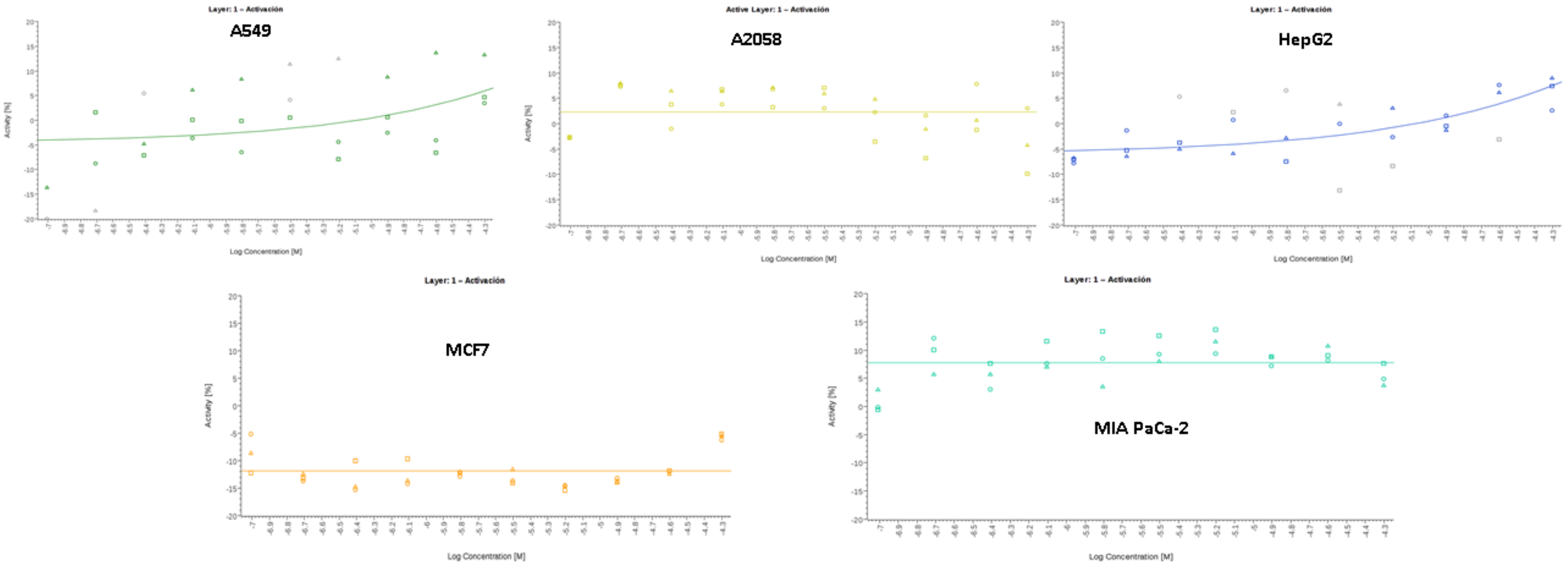

Figure S20. Growth inhibition curves of pipecolisporin (**1**) against human cell lines.

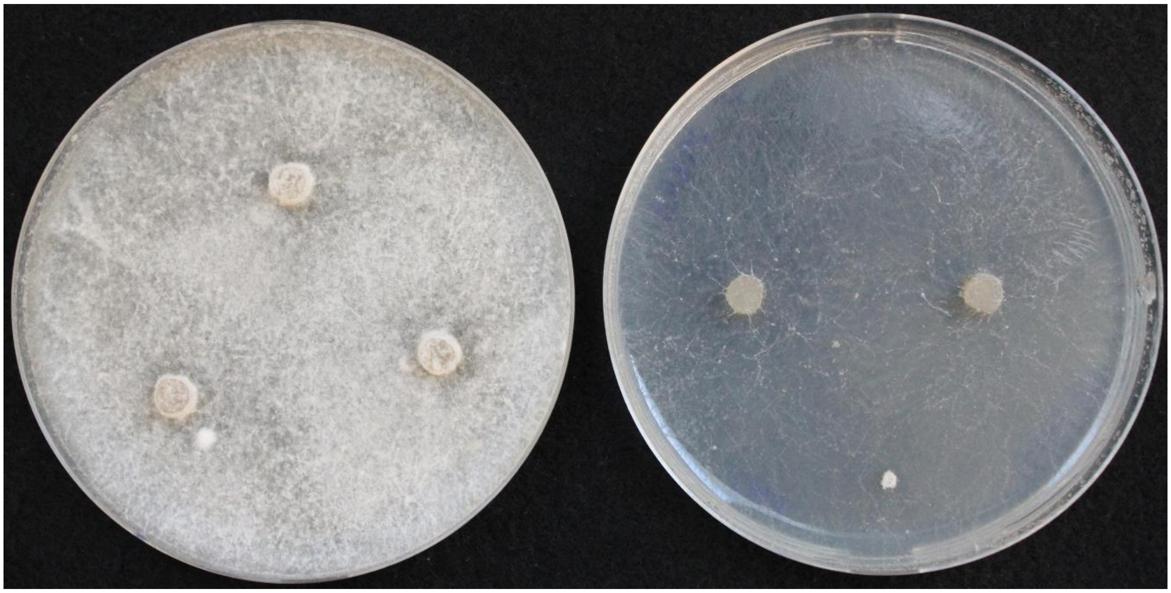

Figure S21. *Nigrospora oryzae* CF-298113. Upper surface overview of a 21 days culture after inoculation on a) Yeast-Malt medium; b) cornmeal medium.

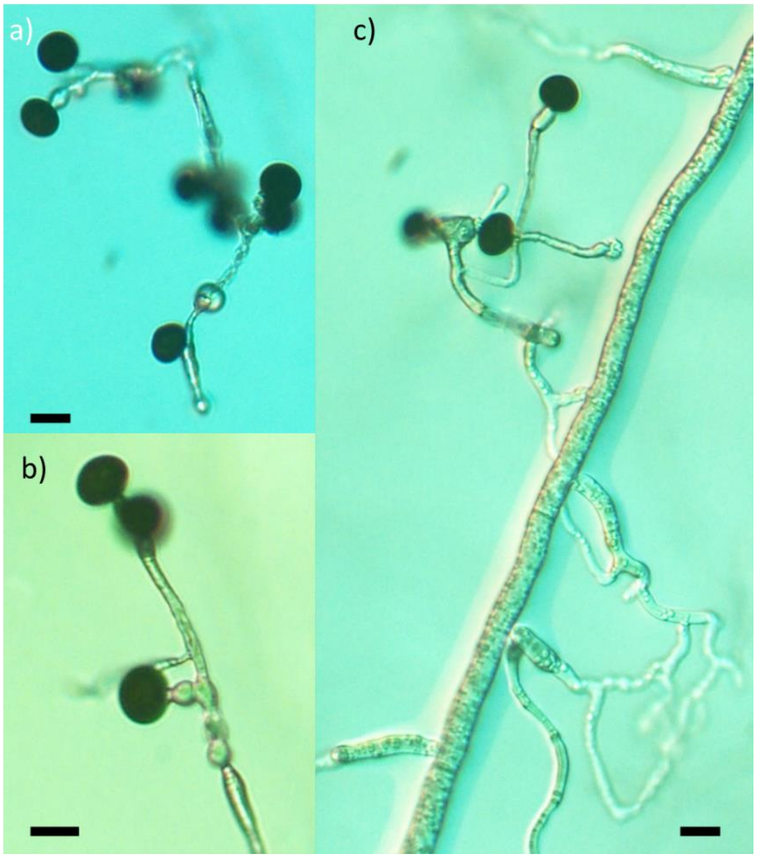

Figure S22. Conidiogenous cells giving rise to conidia. Scale bars: a-c = 10 μm.
